# Previous Vitamin D Supplementation and Morbidity and Mortality Outcomes in People Hospitalised for COVID19: A Cross-Sectional Study

**DOI:** 10.3389/fpubh.2021.758347

**Published:** 2021-09-24

**Authors:** Juan Antonio Arroyo-Díaz, Josep Julve, Bogdan Vlacho, Rosa Corcoy, Paola Ponte, Eva Román, Elena Navas-Méndez, Gemma Llauradó, Josep Franch-Nadal, Pere Domingo, Didac Mauricio

**Affiliations:** ^1^Infectious Diseases, Department of Medicine, Hospital de la Santa Creu i Sant Pau, Barcelona, Spain; ^2^Institut de Recerca de l'Hospital de la Santa Creu i Sant Pau, Barcelona, Spain; ^3^Diabetis des de l'Atenció Primària (DAP)-Cat Group, Unitat de Suport a la Recerca Barcelona, University Institute for Primary Care Research (Institut Universitari per a la recerca a l'Atenció Primària (IDIAP) Jordi Gol), Barcelona, Spain; ^4^Center for Biomedical Research in the Network in Bioengineering, Biomaterials, and Nanomedicine, Madrid, Spain; ^5^Department of Endocrinology and Nutrition, Hospital de la Santa Creu i Sant Pau, Barcelona, Spain; ^6^Department of Medicine, Faculty of Medicine, Autonomous University of Barcelona, Barcelona, Spain; ^7^Department of Medicine, Hospital de la Santa Creu i Sant Pau, Barcelona, Spain; ^8^Unitat de Suport a la Recerca Barcelona, University Institute for Primary Care Research (IDIAP Jordi Gol), Barcelona, Spain; ^9^Department of Endocrinology and Nutrition, Hospital del Mar, Parc de Salut Mar, Barcelona, Spain; ^10^Centro de Investigación Biomédica en Red (CIBER) of Diabetes and Associated Metabolic Diseases, Instituto de Salud Carlos III, Madrid, Spain; ^11^Primary Health Care Center Raval Sud, Gerència d'Atenció Primaria, Institut Català de la Salut, Barcelona, Spain; ^12^Departament of Medicine, Universitat de Vic - Universitat Central de Catalunya, Barcelona, Spain

**Keywords:** COVID-19, hospitalisation, mortality, invasive mechanic ventilation, vitamin D

## Abstract

**Aim:** The study aim was to assess the association of vitamin D supplementation before hospital admission and severe outcomes in subjects admitted for COVID-19.

**Methods:** We performed a cross-sectional analysis of pseudonymised medical record data from subjects admitted to the Hospital de la Santa Creu i Sant Pau (Barcelona, Spain) for COVID-19 during March and April 2020. The composite primary study outcome was defined as death and/or invasive mechanical ventilation (IMV). Association between risk factors and study outcomes was evaluated by bivariate analysis, followed by logistic regression analysis.

**Results:** In total, 1,267 persons were hospitalised during the observation period. Overall, 14.9% of the subjects were on active vitamin D supplementation treatment before admission. The subjects in the vitamin D group were significantly older than subjects without vitamin D supplementation. We observed higher rates of the primary outcome (death and/or IMV) among the persons with previous use of vitamin D (30.1 vs. 22.9% in those not receiving treatment). In the bivariate analysis, previous use of vitamin D was positively associated with death and/or IMV [odds ratio (OR): 1.45 95% CI: 1.03; 2.04]; however, after adjustment for other risk factors this association disappeared (OR: 1.09 95%CI: 0.65; 1.81).

**Conclusion:** We did not find an association between vitamin D supplementation before hospital admission and death and/or IMV in subjects admitted for COVID-19. The age and the burden of age-associated comorbidities were independently associated with the in-hospital events.

## Introduction

From the start of the COVID-19 pandemic to July 2021, more than 191,158,708 new cases and ~4.2 million of deaths have been reported worldwide (1). Although several different vaccines are in use, the number of COVID-19 cases globally remains high, and deaths continue to increase (2). The United States and the European Union drug regulatory agencies have already authorised new indications for treatments such as remdesivir, lopinavir/ritonavir and interferon for subjects hospitalised with COVID-19. However, little or no effect on overall mortality, initiation of ventilation and duration of hospital stay was observed in the corresponding clinical trials (3). In the RECOVERY clinical trial, dexamethasone reduced the mortality among subjects hospitalised for COVID-19 receiving either invasive mechanical ventilation (IMV) or oxygen alone (4). During the pandemic, different therapies have been explored to prevent or treat the disease, including the use of vitamin D supplementation.

Vitamin D is a fat-soluble vitamin that is either formed in the skin or ingested in the diet; this vitamin enters the bloodstream and travels to the liver and kidney where it is hydroxylated on carbons 25 and 1 to form 25-hydroxyvitamin D (25(OH)D) and 1,25-dihydroxy vitamin D (1,25(OH)D), respectively (5). The latter is the active hormone with a wide range of effects. Both children and adults are at risk of developing vitamin D deficiency, which is very common globally. For instance, in Spain 33.9% of the population is at risk for vitamin D deficiency (6).

In addition to its actions on calcium absorption and bone mineralization, this vitamin has pleiotropic actions, including the regulation of immune responses and inflammation. It has been hypothesised that its deficiency can diminish immune responses to respiratory viruses (7). A favourable effect of vitamin D supplementation on the Acute Respiratory Distress Syndrome (ARDS) has been reported, which may be elicited through activation of the vitamin D receptor (VDR) signalling pathway, and a consequential decrease of cytokine/chemokine hypersecretion, modulation of the activity of neutrophils and preservation of the integrity of the pulmonary epithelial barrier (8). In addition, vitamin D has been implicated in preventing or improving adverse outcomes of COVID-19 by activating or repressing genes *via* the VDR that are involved in regulating the renin-angiotensin system (RAS) or innate and adaptive cellular immunity (9).

The effects of vitamin D in preventing COVID-19 infection have been addressed in a few observational studies with discordant observations. An initial observational study reported that vitamin D deficiency was associated with an increased risk of acquiring COVID-19 infection (10, 11). Similar findings were observed in two other observational studies from the US. In one of them, the probability of testing positive for COVID-19 increased with decreasing levels of vitamin D among black women (12), while in the other among black individuals, lower levels of vitamin D were associated with increased risk of COVID-19 (13). Recently, the results of two meta-analyses have been reported. One of them included a total of 29 observational studies, with exclusion of those without recent measurement of vitamin D levels; this meta-analysis found lower levels of vitamin D among subjects with an active COVID-19 infection and also among those with severe COVID-19 (14). However, in the other meta-analysis that included 31 observational studies, the authors did not find a significant association between vitamin D deficiency and COVID-19 health outcomes (15). It should be underlined that the design of these observational studies does not allow a cause-effect relationship between vitamin D status and the risk of COVID-19 or the outcomes of the disease. Regarding the relationship between vitamin D status and COVID-19 outcomes, several observational studies found that patients with COVID-19 requiring hospitalisation or those with severe disease had lower vitamin D concentration or vitamin D deficiency (16–19). In this respect, a systematic review and meta-analysis of 39 studies concluded that despite high heterogeneity and methodological differences, there was a relationship between low 25 (OH) D concentrations and SARS-CoV-2 infection, severity of the disease, and mortality (20). However, the authors concluded that further studies should investigate the association between vitamin D and COVID-19, especially in subgroups of age and sex (20). Moreover, two quasi-experimental studies and a pilot randomised clinical trial reported that vitamin D3 supplementation was associated with less severe COVID-19 (7, 8, 21).

As we are in need of further evidence on the association of vitamin D and COVID-19 outcomes, we undertook the current study with the aim of assessing the association of vitamin D supplementation prior to hospital admission and adverse outcomes in subjects admitted for COVID-19.

## Methods

### Study Design and Settings

We analysed cross-sectional data from hospitalised individuals infected with SARS-CoV-2, stratified by previous vitamin D supplementation before hospital admission. Data were obtained from an anonymised electronic health records database from the Hospital de la Santa Creu i Sant Pau (Barcelona, Spain). The database included retrospective clinical information, information on admission, diagnostic and procedure codes, prescribed medications, and laboratory parameters from 1,267 subjects admitted between March and April 2020. Outcome events were documented from hospital admission until discharge or death.

The study was approved by the Ethics Committee of the Hospital de la Santa Creu i Sant Pau (Re. Nr. HSCSP-20/117).

### Inclusion and Exclusion Criteria

The study enrolled subjects between 19 and 101 years with a confirmed PCR SARS-CoV-2 diagnostic test [asymmetric reverse-transcription polymerase chain reaction (RT-PCR) of ART] as documented in the medical record.

### Study Variables

On admission, the following baseline variables were collected: age, sex, smoking status, information on comorbidities recorded in the electronic records [hypertension, hyperlipidaemia, obesity, diabetes, cardiovascular disease (CVD), heart failure, chronic kidney disease (CKD), chronic obstructive pulmonary disease (COPD), and the Charlson comorbidity index], and blood laboratory parameters (i.e., lymphocytes, C-reactive protein, interleukin-6, ferritin, D-dimer, lactate dehydrogenase, PaO_2_, and the PaO_2_/FiO_2_ index). Vitamin D exposure was defined as a current prescription of any form of active vitamin D supplement at the time of admission to the hospital.

Outcome events during the hospital stay were predefined as: death, admission to the intensive care unit (ICU), days of ICU stay, hospitalisation duration, and invasive mechanical ventilation (IMV). The primary study outcome was predefined as death and/or IMV.

### Statistical Methods

We analysed demographic and clinical characteristics according to vitamin D supplementation prior to admission. Quantitative variables were summarised according to their distribution [median, first and third quartile or mean and standard deviation (±SD)] and or categorical variables as frequency, number and percent (%). The association between events (mechanical ventilation, mortality, and mortality or mechanical ventilation) and previous use of vitamin D was evaluated by bivariate analysis, followed by logistic regression analysis adjusted for sex and age and associated risk factors (age, sex, obesity, hypertension, diabetes, hyperlipidaemia, CVD, CKD, COPD, cancer, Charlson comorbidity index and PaO_2_/FiO_2_ index). Several models of interest were tested, with the sequential inclusion of different covariates and the estimated differences expressed as adjusted odds ratio (AOR) and their respective 95% confidence intervals (CI). Additionally, we performed the same bivariate analysis followed by logistic regression analysis (using the same previously mentioned risk factors) for a subgroup of subjects who were 60 years of age or older. Data management and statistical analyses were performed using the R statistical software version 3.6.1 (https://www.r-project.org/).

## Results

During March–April 2020 (corresponding to the first COVID wave), 1,267 persons were hospitalised due to COVID-19 at the Hospital de la Santa Creu i Sant Pau. One hundred and eighty-nine (14.9%) subjects were receiving active vitamin D supplementation on admission (Vitamin D group). Supplementary Figure 1 presents the flowchart of the study.

### Baseline Characteristics

The baseline clinical characteristics of the study subjects are shown in Table 1. The mean age of subjects was 64.7 years, and the majority were male (54.9%). However, we observed differences between the groups for age and sex, whereby subjects in the vitamin D group were older and were more frequently female. Subjects in the vitamin D group had a higher rate of most concomitant diseases of interest, except for obesity, which was more frequent in the group without vitamin D. Regarding the laboratory parameters, the majority of the parameters (lymphocytes, C-reactive protein, interleukin 6, D-dimer, Blood lactate, PaO_2_, and PaO_2_/FiO_2_ Index) were higher in the vitamin D group, however, no statistically significant differences were observed between the groups, except for PaO_2_ where borderline differences were observed.

**Table 1:** Baseline characteristics of all subjects and according to vitamin D supplements.

**Characteristic**	**All subjects ***N*** = 1,267**	**Group not receiving vitamin D supplements**	**Group receiving vitamin D supplements**	***p*-value**
		***N*** **= 1,078**	***N*** **= 189**	
Age, mean (SD), years	64.7 (16.3)	63.2 (16.3)	73.3 (13.7)	<0.001
Sex (male), *n* (%)	696 (54.9)	634 (58.8)	62 (32.8)	<0.001
Smoking, *n* (%)	129 (10.2)	111 (10.3)	18 (9.52)	0.76
**Comorbidities**, ***n*** **(%)**				
Hypertension	602 (47.5)	484 (44.9)	118 (62.4)	<0.001
Hyperlipidaemia	509 (40.2)	404 (37.5)	105 (55.6)	<0.001
Obesity	567 (44.8)	488 (45.3)	79 (41.8)	0.38
Diabetes	252 (19.9)	205 (19.0)	47 (24.9)	0.07
Cardiovascular diseases	152 (12.0)	118 (10.9)	34 (18.0)	0.01
Heart failure	107 (8.45)	80 (7.42)	27 (14.3)	0.003
Atrial fibrillation	126 (9.94)	100 (9.28)	26 (13.8)	0.07
Chronic kidney disease	155 (12.2)	105 (9.74)	50 (26.5)	<0.001
COPD	219 (17.3)	179 (16.6)	40 (21.2)	0.13
Charlson comorbidity Index (>2)	687 (54.2)	525 (48.7)	162 (85.7)	<0.001
**Laboratory parameters, mean (SD)**				
Lymphocytes (μL)	1,000 (2,000)	970 (790)	1,200 (4,900)	0.28
C-reactive protein (mg/L)	145 (110)	145 (111)	148 (109)	0.72
Interleukin 6 (pg/mL)	293 (752)	292 (775)	300 (412)	0.97
Ferritin (μg/L)	1,832 (6,115)	1,840 (6,473)	1,789 (3,440)	0.93
D-dimer (ng/mL)	6,200 (1,7900)	5,900 (18,000)	7,400 (17,000)	0.34
Blood lactate (mmol/L)	1.11 (1.23)	1.09 (1.09)	1.22 (1.87)	0.24
PaO_2_ (mmHg)	77.0 (38.2)	76.0 (34.7)	82.9 (54.7)	0.046
PaO_2_/FiO_2_ (mmHg)	283 (120)	284 (121)	280 (110)	0.77

*COPD, chronic obstructive pulmonary disease; SD, standard deviation*.

### Events During in-hospital Stay

The events and complications during the hospital stay are presented in Table 2. A total of 217 (17.1%) subjects died during in-hospital stay. We observed statistically significant differences between groups in the primary study outcome (death and/or IMV) and for death: both events were more frequent among subjects with previous use of vitamin D (22.9 vs. 30.1% and 15.5 vs. 26.5%, respectively). However, the percentage of subjects admitted to the intensive care unit (ICU), days in ICU, as well as duration of hospitalisation was lower in the vitamin D group.

**Table 2:** Intra hospital events overall and according to Vitamin D supplements in the whole study population and in the subgroup of 60 years or more.

	**All**			**≥60 years**		
	**All subjects**	**Group not receiving Vitamin D supplements**	**Group receiving vitamin D supplements**	**All subjects**	**Group not receiving vitamin D supplements**	**Group receiving vitamin D supplements**
	***N*** **= 1,267**	***N*** **= 1,078**	***N*** **= 189**	***N*** **= 797**	***N*** **= 638**	***N*** **= 159**
Death, *n* (%)	217 (17.1)	167 (15.5)*	50 (26.5)*	201 (25.2)	154 (24.1)	47 (29.6)
IMV, *n* (%)	124 (9.8)	113 (10.5)	11 (5.8)	93 (11.7)	85 (13.3)	8 (5.0)
Death and/or IMV, *n* (%)	304 (23.9)	247 (22.9)*	57 (30.1)*	262 (32.8)	211 (33.0)	51 (32.0)
ICU, *n* (%)	146 (11.5)	133 (12.3)	13 (6.9)	104 (13.0)	95 (14.9)	9 (5.7)
Days in ICU, mean (SD)	1.87 (7.1)	2.00 (7.4)	1.08 (5.2)	2.32 (7.9)	2.65 (8.4)	1.01 (5.3)
Hospitalisation duration (days), mean (SD)	8.75 (10.4)	8.91 (10.7)	7.86 (8.5)	9.17 (11.5)	9.56 (12.2)	7.64 (8.1)

*ICU, intensive care unit; IMV, invasive mechanical ventilation*.

* *p-value < 0.05*.

The sub-analysis of subjects over 60 years showed a slightly higher percentage for the death and/or IMV in the group receiving vitamin D. However, no statistically significant differences for the primary outcome were observed between groups in this subgroup.

### Factors Associated With Events by Previous Use of Vitamin D

**Figure 1:**
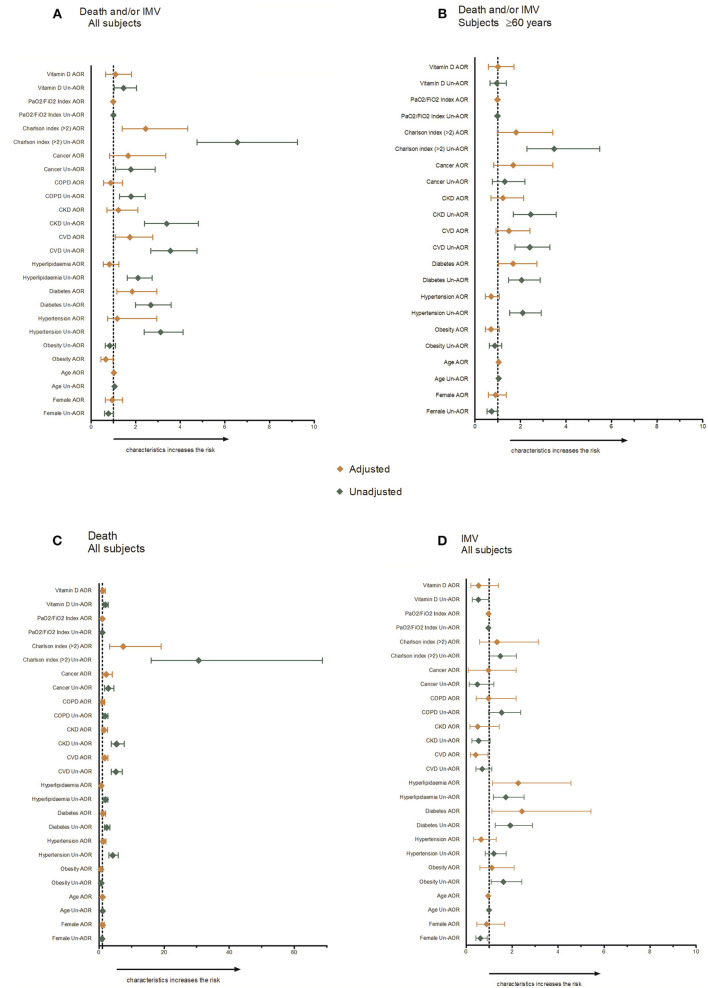
Risk factors for severity outcomes.

The bivariate and logistic regression analysis results for main composite outcome are presented in Supplementary Table 1 and Figure 1A. In the bivariate analysis, we observed that vitamin D and most of the risk factors and co-morbidities (older age, hypertension, diabetes, hyperlipidaemia, CVD, CKD, COPD, cancer, higher Charlson comorbidity index score) were positively associated with the composite primary outcome (death and/or IMV). We observed negative associations only for the PaO_2_/FiO_2_ Index and female sex.

In the logistic regression analyses, after adjustment for all associated risk factors, only age, diabetes, CVD, and a higher Charlson comorbidity index score were positively associated with the primary outcome. We did not observe a significant association between previous use of vitamin D and death and/or IMV (AOR: 1.09, 95% CI: 0.65; 1.81). Obesity and PaO_2_/FiO_2_ index were negatively associated with the main composite outcome in this analysis.

The results for the separate events (death and IMV) in all subjects are presented in Supplementary Table 1 and Figures 1C,D. Similar associations were observed in the bivariate analysis for mortality, except for obesity, where a negative association with death was observed. For the IMV outcome, obesity was positively associated, while vitamin D supplementation was negatively associated. No associations were observed between death and/or IMV and vitamin D use when the model was adjusted for all factors.

### Factors Associated With Events by Previous Use of Vitamin D Among Persons at Least 60 Years or Older

Supplementary Table 2 and Figure 1B show the bivariate and logistic regression analyses for persons at least 60 years or older. In subjects aged 60 years or older, the same factors (age, hypertension, diabetes, CVD, CKD, cancer, a higher Charlson comorbidity index score) were positively associated with the primary outcome (death and/or IMV), although with a lower unadjusted OR. In the adjusted logistic regression analyses, only age and diabetes were positively associated with the main outcome.

## Discussion

The results of the present cross-sectional study of 1,267 persons infected with COVID-19 during March and April 2020 showed higher in-hospital mortality and/or IMV among subjects treated with vitamin D prior to hospital admission in the crude analysis. However, no significant association between use of vitamin D supplementation prior to hospitalisation and risk of death and/or IMV was observed in the fully adjusted model. Instead, age and the burden of age-associated comorbidities were independently associated with the in-hospital events.

The importance of vitamin D on the clinical severity of COVID-19 remains controversial. So far, the studies published on this topic are heterogeneous in their methodology and the findings obtained. Some studies support the association between vitamin D and favourable disease outcomes, whereas others did not observe significant associations. For example, in an observational study from Spain with 216 hospitalised subjects with COVID-19, the researchers did not observe an association between vitamin D status (circulating levels of 25OHD) and the severity of COVID-19 infection (ICU admission, the need for mechanical ventilation, or mortality); these results are in line with the results observed in our study where we did not find any signal for an association (17). Despite the small number of subjects (only 19) identified as receiving vitamin D supplementation in that study, similarities could be observed in terms of demographic and clinical characteristics with subjects included in current study, such as higher percentage of women and more severe comorbidity profile (hypertension, cardiovascular diseases) compared with COVID-19 subjects not receiving vitamin D supplementation. Moreover, the authors reported lower percent of persons with PaO_2_/FIO_2_ ratio <300, lower median serum ferritin and less need for tocilizumab therapy among the persons with oral vitamin D supplements (17). In contrast, we did not observe any differences in the PaO_2_/FIO_2_ ratio and ferritin levels between our study groups. Moreover, in the logistic regression models, PaO_2_/FIO_2_ ratio and obesity were associated with the study outcomes. These findings are in line with other previously published reports. The PaO_2_/FIO_2_ ratio has been reported as an independent factor related to death in COVID-19 subjects receiving intensive care (22). In addition, obesity has been associated with substantially increased risks of severe outcomes in patients with COVID-19 (23). Therefore, it is perhaps surprising that in the logistic regression models, we found that the obesity was negatively associated with the study outcomes. This could be due to the J-shaped association between BMI and the mortality curve. It was already reported that survival rates of subjects with moderate obesity were higher than for those with normal body mass index (BMI), overweight or severe obesity subjects (23). However, a clear limitation of our study is that data on BMI were not available and the analyses were only based on a clinical recorded diagnosis of obesity in the clinical records, which is not a solid variable to analyse the contribution of adiposity on the outcomes.

So far, regarding studies that tested the use of vitamin D as a preventive therapy for COVD-19, one pilot randomised clinical trial in Spain with 76 participants reported that admission to the ICU was reduced in the group receiving a high dose of vitamin D (7). Two additional quasi-experimental studies from France observed that only regular use of vitamin D was associated with less severe COVID-19 and a lower mortality rate (9, 21), while the administration of vitamin D after COVID-19 diagnosis did not affect severity outcomes (9). It is important to mention that the authors in the French study had full information on supplemental vitamin D exposure (dose and duration of supplementation) (9), which was not the case of our study, resulting again in another important limitation.

Several systematic reviews and meta-analyses have been published on this topic as well. In a systematic review including 4 observational studies to assess the association between vitamin D supplementation or level with COVID-19 infection susceptibility and outcomes (clinical course, morbidity and mortality outcomes), no robust association was found between vitamin D and COVID-19 severity of symptoms or mortality (24). Another systematic review with 11 studies about vitamin D levels/supplementation and risk of COVID-19 infection, adverse outcomes and possible benefits among subjects aged 60 years or over concluded that supplementation with vitamin D during COVID-19 reduced the risks for mortality, high flow oxygen therapy needs, or ICU support (25). Nevertheless, the authors pointed out the importance of the characteristics of the supplementation regimen, with aspects that remain unresolved such as the dose, frequency of administration, and duration (25). In our sub-analysis of subjects aged 60 years or over, the composite outcome of death and/or IMV was more frequent for both groups (on vitamin D and without) than in the total study group; however, no differences were observed regarding the use of previous vitamin D. Independently of previous vitamin D intake, evidence suggests that subjects over 60 years are more prone to severe complications and longer duration of COVID-19 compared with younger persons (age 60 or younger) (26–28); this was also observed in our study. Subjects on vitamin D in the current study were 10 years older compared with subjects without vitamin D supplementation. A recent meta-analysis of three randomised controlled trials found no effect of vitamin D supplementation in participants with COVID-19 for all-cause mortality (an outcome in two of the studies), and the need for invasive mechanical ventilation and adverse events (one of the studies) (29). Moreover, the authors reported high inconsistency in reporting of adverse reactions or safety of vitamin D and a lack of evidence that this supplementation prevented death from COVID-19 (29).

Based on the currently published observational studies, vitamin D deficiency seems to be associated with an increased risk of severe disease and mortality from COVID-19 (16–20, 22, 23, 25, 30). In a randomised clinical trial from Brazil, that included patients already hospitalised with moderate to severe COVID-19 in whom a single high dose of vitamin D was administrated, the authors reported that it was too early to conclude whether vitamin D supplementation may improve the disease prognosis (31). Further, treatment with a high dose of vitamin D in subjects who have already presented with complications of COVID-19 may not be effective since the organ damage by virus had already initiated. The inverse relationship between the risk of acute respiratory infections and vitamin D blood levels is well-reported (7, 10, 32, 33). The possible biological explanation for this effect could be the influence of genes regulating the immune system and the inflammatory response, which might modify the susceptibility to and severity of infections (34). Vitamin D deficiency is highly prevalent, especially in older adults, mainly due to the decreased synthesis in the skin or deficient dietary intake (35), and it could be easily prevented with regular supplementations (25). In our study, another important limitation is the lack of information on the levels of vitamin D among the study subjects. Our data suggest that the main drivers of severe outcomes in COVID-19 are age-associated comorbidities. Since most of our subjects taking vitamin D were older adults with a high burden of comorbidities, the potential benefits of vitamin D supplementation may be very difficult to demonstrate. Additionally, it is possible that in many of these subjects the reason for prescription of vitamin D supplementation was a status of vitamin D deficiency.

Apart from those already stated, this study has additional limitations. The database was created from anonymised, routinely collected medical data of hospitalised subjects during the first wave of the pandemic; therefore, the amount of incomplete data was high. Consequently, some important clinical variables were not considered in the analysis, such as BMI, dose and time of exposure related to pharmacological treatment, completeness of information on medical conditions or procedures prior to admission. Moreover, we did not have variables related to vitamin D such as duration of vitamin D supplementation, doses, adherence to supplementation or blood levels of vitamin D. It should be pointed out that the vitamin D intake in Spain is among the lowest compared with other European countries (36). According to the SEIOMM (Spanish Society for Research on Bone and Mineral Metabolism), the recommendation on the dose and duration of the supplementation with vitamin D should be based on the levels of vitamin D in the given subject. In subjects from the general population with severe deficiency (<10 ng/mL) and a target level of vitamin D >25 ng/mL, the recommended dose is either 16.000 IU/weekly during 5 weeks for calcifediol or 50.000 IU/weekly during 4–6 weeks for cholecalciferol. In those with vitamin D insufficiency (10–25 ng/mL), the recommended dose for cholecalciferol is 25.000 IU/daily and 16.000 IU/monthly for calcifediol (37). Since the group receiving vitamin D supplements in our study consisted mainly of elder subjects (mean age 73.3 years) and predominantly of female sex (67.2%), we assume that the dose of vitamin D prescribed by the treating physician was probably in the range recommended by guidelines. Since this is a cross-sectional study, the logistic regression models should be interpreted with caution; accordingly they only describe the possible association between the risk factors and the studied outcomes.

## Conclusions

No association between vitamin D supplementation prior to hospital admission and important outcomes, i.e., death and/or IMV, or secondary in-hospital events was observed in this cross-sectional study of inpatients with COVID-19. Well-designed randomised clinical trials are needed to confirm the potential role of vitamin D on outcomes of COVID-19.

## Data Availability Statement

The data analysed in this study is subject to the following licences/restrictions: The data controller for Hospital de la Santa Creu i Sant Pau does not allow the sharing of raw data. Requests to access these datasets should be directed to Pere Domingo, pdomingo@santpau.cat.

## Ethics Statement

The studies involving human participants were reviewed and approved by the Ethics Committee of the Hospital de la Santa Creu i Sant Pau (Re. Nr. HSCSP-20/117). Written informed consent for participation was not required for this study in accordance with the national legislation and the institutional requirements.

## Author Contributions

JA-D, JJ, JF-N, PD, and DM: conceptualisation. EN-M: formal analysis. JA-D, DM, PD, ER, and PP: resources and data curation. BV: writing—original draught preparation. BV, RC, JA-D, GL, JF-N, PD, and DM: writing—review and editing. DM and JF-N: supervision. JA-D: project administration. All authors contributed to the article and approved the submitted version.

## Conflict of Interest

RC has received advisory and/or speaking fees from Abbott, Ascensia, Lilly, MSD, Novo Nordisk and Sanofi. JF-N has received advisory and or speaking fees from Astra-Zeneca, Ascensia, Boehringer Ingelheim, GSK, Lilly, MSD, Novartis, Novo Nordisk, and Sanofi; they received research grants to the institution from Astra-Zeneca, GSK, Lilly, MSD, Novartis, Novo Nordisk, Sanofi, and Boehringer. PD has received lecture and Advisory Board fees from Gilead Sciences, Roche, Merck, Sharp and Dohme, ViiV Healthcare, Janssen and Cilag, Theratechnologies, Boehringer Ingelheim, and Ferrer International. PD has received research grants from Gilead Sciences, ViiV Healthcare, GSK, Janssen and Cilag, and Boehringer Ingelheim. DM has received advisory and/or speaking fees from Astra-Zeneca, Ascensia, Boehringer Ingelheim, GSK, Lilly, MSD, Novartis, Novo Nordisk, and Sanofi; they received research grants to the institution from Astra-Zeneca, GSK, Lilly, MSD, Novartis, Novo Nordisk, Sanofi, and Boehringer. The remaining authors declare that the research was conducted in the absence of any commercial or financial relationships that could be construed as a potential conflict of interest.

## Publisher's Note

All claims expressed in this article are solely those of the authors and do not necessarily represent those of their affiliated organizations, or those of the publisher, the editors and the reviewers. Any product that may be evaluated in this article, or claim that may be made by its manufacturer, is not guaranteed or endorsed by the publisher.
