# Supplementary material for: Previous Vitamin D Supplementation and Morbidity and Mortality Outcomes in People Hospitalised for COVID19: A Cross-Sectional Study
Source: Front Public Health. 2021 Sep 24;9:758347. doi: 10.3389/fpubh.2021.758347 (PMC8498099; doi:10.3389/fpubh.2021.758347)
Supplement: Supplementary file 1 [file Data_Sheet_1.pdf]

ONLINE-ONLY SUPPLEMENTARY MATERIALS

These supplemental materials have been provided by the authors to give the readers additional information about the study.

Previous vitamin D supplementation and morbimortality outcomes in people hospitalised for COVID19: a cross-sectional study

Juan Antonio Arroyo-Díaz, Josep Julve , Bogdan Vlacho, Rosa Corcoy, Paola Ponte, Eva Román, Elena Navas-Méndez, Gemma Llauradó, Josep Franch-, Pere Domingo, and Dídac Mauricio.

TABLE OF CONTENTS

		PAGE
**Supplementary Figure 1.**	Study flowchart	2
**Supplementary Table 1**	Un-adjusted and adjusted odds ratios for the study outcomes for all of the subjects	3
**Supplementary Table 2**	Un-adjusted and adjusted odds ratios for main composite outcome Death and/or IMV for Subjects ≥ 60	4

Supplementary Figure 1. Study flowchart

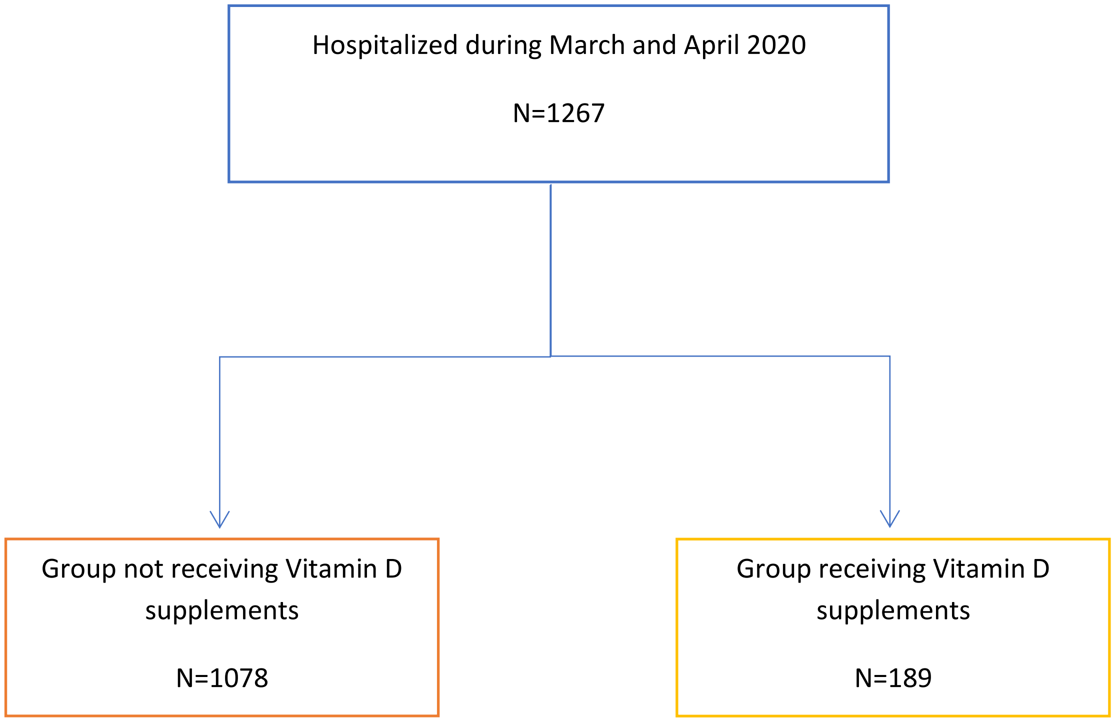

Supplementary table 1. Un-adjusted and adjusted odds ratios for the study outcomes for all study subjects

Variables	Death and/or IMV		Death		IMV	
	Un-adjusted OR (95% CI)	Adjusted OR (95% CI)	Un-adjusted OR (95% CI)	Adjusted OR (95% CI)	Un-adjusted OR (95% CI)	Adjusted OR (95% CI)
Vitamin D (yes), ref: no	1.45* (1.03; 2.04)	1.09 (0.65; 1.81)	1.96* (1.36;2.81)	1.15 (0.70; 1.89)	0.53* (0.27;0.97)	0.54 (0.20; 1.40)
Sex (Female), ref: male	0.78 (0.60; 1.01)	0.95 (0.64; 1.41)	0.92 (0.68;1.23)	1.06 (0.69; 1.63)	0.62 (0.42;0.91)	0.88 (0.46; 1.68)
Age	1.05* (1.04; 1.06)	1.02* (1.01; 1.04)	1.08* (1.07;1.10)	1.05* (1.03; 1.07)	0.99* (0.98;1.00)	0.96* (0.93; 0.98)
Obesity (yes), ref: no	0.83 (0.63; 1.09)	0.66* (0.44; 0.97)	0.64*(0.46;0.87)	0.59* (0.38; 0.89)	1.62* (1.09;2.42)	1.12 (0.60; 2.08)
Hypertension (yes), ref: no	3.12* (2.38; 4.12)	1.17 (0.74; 1.84)	4.19* (3.03;5.88)	1.31 (0.80; 2.16)	1.20 (0.83;1.74)	0.65 (0.32; 1.30)
Diabetes (yes), ref: no	2.67* (1.99; 3.59)	1.84* (1.16; 2.94)	2.40* (1.73;3.32)	1.24 (0.78; 1.96)	1.92* (1.26;2.88)	2.42* (1.11; 5.43)
Hyperlipidemia (yes), ref: no	2.10* (1.62; 2.73)	0.82 (0.54; 1.24)	2.01* (1.49;2.70)	0.69 (0.44; 1.07)	1.73* (1.19;2.52)	2.26* (1.15; 4.56)
CVD (yes), ref: no	3.56* (2.67; 4.74)	1.73* (1.08; 2.76)	5.18* (3.79;7.09)	1.75* (1.12; 2.75)	0.70 (0.42;1.12)	0.42* (0.18; 0.95)
CKD (yes), ref: no	3.39* (2.39; 4.80)	1.22 (0.70; 2.10)	5.40* (3.76;7.75)	1.56 (0.94; 2.59)	0.54 (0.25;1.04)	0.50 (0.16; 1.44)
COPD (yes), ref: no	1.77* (1.28; 2.42)	0.88 (0.55; 1.41)	1.89* (1.33;2.67)	1.07 (0.67; 1.70)	1.54 (0.97;2.37)	0.97 (0.43; 2.17)
Cancer (yes), ref: no	1.78*(1.09; 2.86)	1.66 (0.82; 3.34)	2.85* (1.73;4.60)	2.19* (1.17; 4.08)	0.49 (0.14;1.20)	0.38 (0.09; 1.36)
Charlson comorbidity index (>2), ref: ≤2	6.56*(4.74; 9.25)	2.44* (1.39; 4.33)	30.6* (16.0;68.6)	7.40* (3.28; 19.09)	1.48* (1.02;2.19)	1.34 (0.58; 3.15)
PaO2/FiO2 index	0.99*(0.98; 0.99)	0.99*(0.98; 0.99)	0.99* (0.99;0.99)	0.99* (0.99; 0.99)	0.97* (0.97;0.98)	0.97* (0.96; 0.97)
Observations	-	1060	-	1060	-	1060
R2 Tjur	-	0.427	-	0.293	-	0.592

CVD: cardiovascular disease; CKD: chronic kidney disease; COPD: chronic obstructive pulmonary disease; OR: odds ratios ; 95% CI ; 95% confidence intervals; IMV: invasive mechanical ventilation ; * p-value <0.05

Supplementary table 2. Un-adjusted and adjusted odds ratios for main composite study outcome (death and/or IMV) for subjects aged ≥ 60

	Death and/or IMV	
	Subjects≥60	
Variables	Un-adjusted OR (95% CI)	Adjusted OR (95% CI)
Vitamin D (yes), ref: no	0.96 (0.66; 1.38)	1.01 (0.59; 1.71)
Sex (Female), ref: male	0.73 (0.54; 0.98)	0.90 (0.59; 1.38)
Age	1.04* (1.02; 1.06)	1.04* (1.01; 1.06)
Obesity (yes), ref: no	0.87 (0.63; 1.18)	0.71 (0.46; 1.08)
Hypertension (yes), ref: no	2.10* (1.53; 2.91)	1.21 (0.75; 1.96)
Diabetes (yes), ref: no	2.05* (1.48; 2.86)	1.68* (1.04; 2.72)
CVD (yes), ref: no	2.41* (1.76; 3.30)	1.49 (0.92; 2.41)
CKD (yes), ref: no	2.45* (1.68; 3.57)	1.23 (0.70; 2.14)
Cancer (yes), ref: no	1.31 (0.76; 2.20)	1.68 (0.82; 3.43)
Charlson comorbidity index (>2), ref: ≤2	3.48* (2.29; 5.48)	1.81 (0.98; 3.43)
PaO2/FiO2 index	0.99*(0.99; 0.99)	0.99* (0.99; 0.99)
Observations	-	644
R2 Tjur	-	0.354

CVD: cardiovascular disease; CKD: chronic kidney disease; COPD: chronic obstructive pulmonary disease; OR: odds ratios; 95% CI; 95% confidence intervals; IMV: invasive mechanical ventilation; * p-value <0.05
